# Impact of Coronavirus-19 Pandemic and Lockdown on Admissions for Ischemic Heart Disease

**DOI:** 10.26502/fccm.92920270

**Published:** 2022-07-07

**Authors:** Juan Enrique Puche García, Marta Iturregui Guevara, Etelvino Silva García, Raquel Campuzano Ruiz, Rafael Vázquez García

**Affiliations:** 1 Cardiology Department, Universitary Hospital “Puerta del Mar”, Cádiz, Spain; 2 Endocrinology Department, Universitary Hospital from Jerez, Cádiz, Spain; 3 Institute of Biomedical Research and Innovation of Cádiz (INIBICA), Cádiz, Spain; 4 Cardiology Department, Universitary Hospital Alcorcón Foundation, Madrid, Spain

**Keywords:** Lockdown, SARS-CoV-2, Acute coronary syndrome, Ischemic heart disease, Cardiovascular risk factors

## Abstract

**Background:**

In early 2020, the SARS-CoV-2 pandemic caused an unprecedented overload for the health service. A decrease in admissions for Acute Coronary Syndrome (ACS) was reported during lockdown, although many aspects remain to be clarified. The main objective of this study was to evaluate the impact of the pandemic and of lockdown itself in this area.

**Methods:**

We performed a retrospective observational study based on data from patients who visited the emergency department of a tertiary hospital with chest pain during 2018-2020, as well as those who were admitted for ACS. Personal details, date of admission, additional test results (laboratory and echocardiography), and therapy were recorded. Patients were divided into 3 groups: preCOVID (n=1,301), lockdown (n=45), and postlockdown (n=343).

**Results:**

Fewer visits to the emergency department for chest pain and admissions for ACS were recorded during lockdown (48.6% and 51.1% respectively, p<0.05). Patients who were admitted during lockdown were characterized by poorer control of cardiovascular risk factors, visited later (more evolving infarctions: 2.7% vs. 14.3%, p<0.05), experienced more echocardiographic complications during admission, and had more than 3-fold mortality rates (both in-hospital and postdischarge).

**Conclusion:**

The COVID-19 pandemic and lockdown itself had a negative effect on ischemic heart disease beyond SARS-CoV-2 infection.

## 1. Introduction

In early 2020, SARS-CoV-2 infection generated an unprecedented overload for hospitals owing to its exponential incidence and potential morbidity and mortality. On March 11, the World Health Organization declared a state of pandemic with uncertain transmissibility, difficult control, and poor response to initial treatments. According to official data from the Spanish National Statistics Office, the year 2020 in Spain finished with almost 2,000,000 persons infected and more than 50,000 COVID-19 deaths. Even so, this mortality is lower than the more than 53,000 caused by circulatory system diseases during the same period. Despite the decrease in incidence and mortality of acute myocardial infarction during recent decades [1,2], ischemic heart disease (IHD) continues to be the leading cause of death in the world.

The state of emergency that came into force on March 16, 2020 limited the mobility of the population with the aim of curbing the spread of the virus. The period up to May 10 (when phased reopening started) was characterized by marked excess mortality, which was attributed mainly to COVID-19. However, given that some studies have reported a decrease in care for several diseases during this period, the increase reported could be skewed. Specifically, admissions for Acute Coronary Syndrome (ACS) fell considerably during lockdown [3,4]. Many aspects remain to be clarified with respect to the profile of patients admitted with IHD during lockdown compared with previous years and after lockdown. The same is true of care and results for mortality due to cardiovascular disease and disease overall.

The present study aims to provide answers to these questions and to analyze the care administered for chest pain and ACS in the daily clinical practice of a tertiary hospital during these periods.

## 2. Methods

### 2.1. Study design

We present the results of an observational retrospective study based on data from patients who attended the emergency department of a tertiary hospital during the years 2018-2020 with chest pain (or equivalent). Of a total of 10,356 patients who were seen in the emergency department with chest pain, 1,689 were eventually admitted to different departments (cardiology [1,057, 62.6% of admissions], intensive care [415, 24.6%], internal medicine [196, 11.6%], and other [48, 1.2 %]), with a diagnosis of ST-Segment Elevation Myocardial Infarction (STEMI), non–STEMI (NSTEMI), or unstable angina. Epidemiological and personal variables were recorded (sex, age, Cardiovascular Risk Factors [(CVRFs), arterial hypertension, diabetes mellitus, dyslipidemia] and a history of IHD or Chronic Kidney Disease [CKD]), as were time-related variables (day and month of visit to the emergency department). Similarly, data from echocardiograms before and after the ischemic event were analyzed when available. These were used to design a complications score at discharge. One point was assigned to each of the following scenarios: ischemic dilated cardiomyopathy, grade of left ventricular systolic dysfunction (each grade added an additional point), right ventricular systolic dysfunction, ventricular aneurysm, and at least moderate mitral insufficiency, with a maximum of 7 points.

We also studied the number of catheterizations per patient, as well as the need for Percutaneous Coronary Intervention (PCI), length of stay, and, when the outcome was unfavorable, the cause of death. Patients were divided into 3 groups: preCOVID (January 1, 2019 to March 15, 2020, n=1,301), lockdown (March 16 to May 10, 2020, n=45), and postlockdown (May 11 to December 31 2020, n=343). We tried to minimize the amount of bias by intensive searching using multiple search criteria and institutional programs, as well as contacting out-of-hospital health services for out-of- hospital cardiac arrest registration.

This study had 2 objectives: 1) To characterize the clinical-epidemiological profile of ACS in our care area during the preCOVID era (incidence, sociodemographic profile, percentage of revascularizations, length of stay, and mortality); and 2) To evaluate the impact of the COVID-19 pandemic and lockdown itself in this setting. The study fulfilled the stipulations of the Declaration of Helsinki and was approved by the local Research Ethics Committee (registry number 85.21).

### 2.2. Statistical analysis

Categorical variables were expressed as frequency and percentage and compared between groups using Chi-square or Fisher’s test when appropriate. The 95% confidence interval was also calculated. Continuous variables were expressed as mean ± standard deviation, with normal distributions verified by the Lilliefors test or Shapiro-Wilks test according the number of samples and tested by unpaired t test or Mann-Whitney U test, according to normality, and paired data by paired t test or Wilcoxon analysis. One-way ANOVA test or Kruskall-Wallis test according to normality were used to evaluate mean differences in 2 or more groups. Statistical significance was defined at P<0.05. All data were analyzed using the SPSS version. 23.0 statistical package (SPSS, Inc., Chicago, Illinois, USA).

## 3. Results

### 3.1. Care in the emergency department

**Figure 1:**
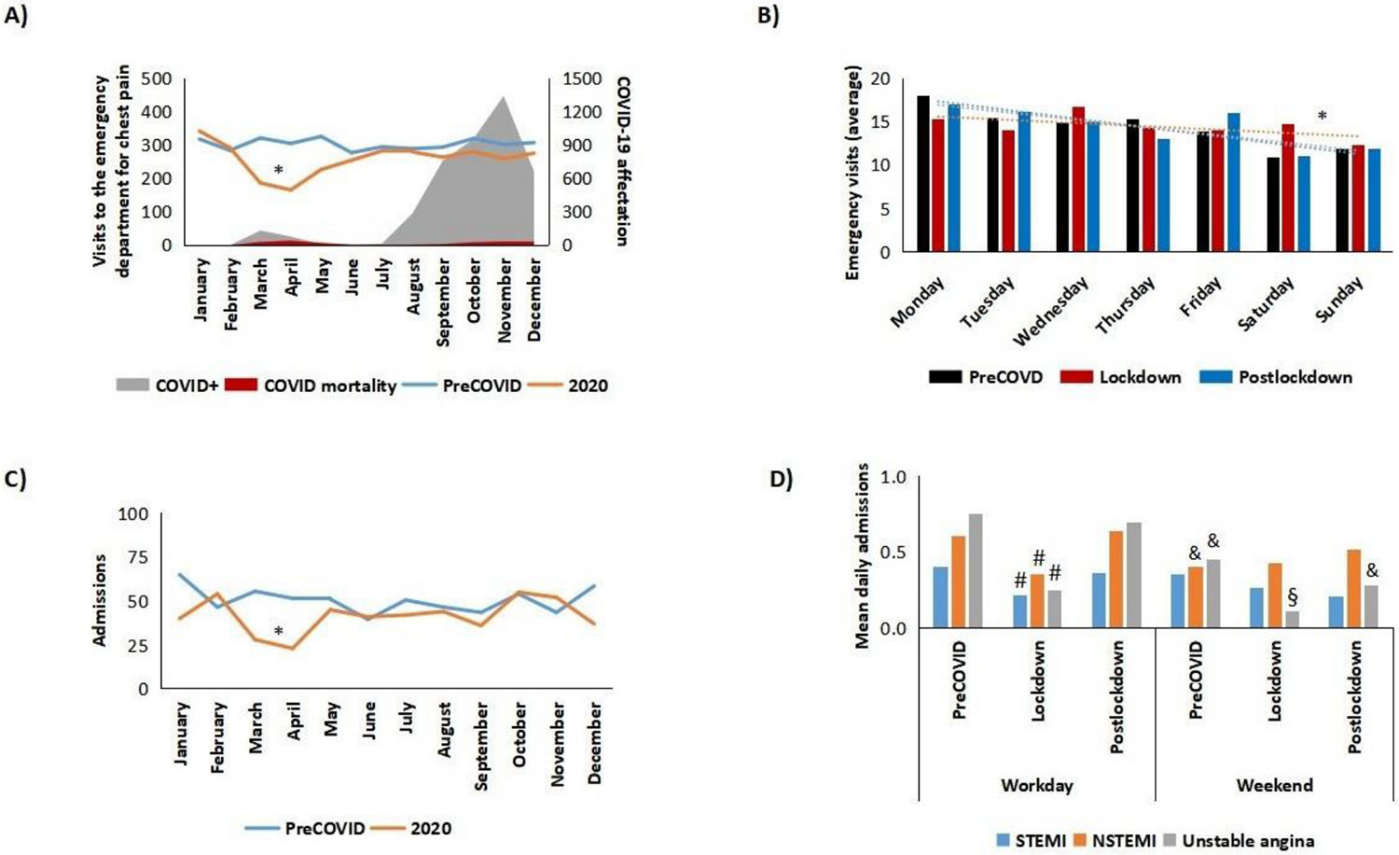
Emergency department visits due to chest pain and admissions for acute coronary syndrome. A) Emergency visits compared to positive cases and mortality by COVID-19 in our sanitary area. B) Daily distribution of visits. C) Monthly distribution of admissions. D) Weekly profile (working day vs. weekends) of admissions for different subtypes of ACS. *p<0.05 vs. preCOVID and postlockdown. #p<0.05 vs. preCOVID and postclockdown working days & p<0.05 vs. preCOVID working days §p<0.05 vs. lockdown working days. NSTEMI, non–ST-Segment Myocardial Infarction; STEMI, ST-Segment Elevation Myocardial Infarction.

In contrast with most regions in Spain, it was not the first wave that led to the highest incidence of cases of or death from COVID-19 in our care area (216 positive cases and 91 deaths, according to official sources), but the second wave (3,711 positive cases and 101 deaths) (Figure 1A). Nevertheless, during lockdown (March 16, 2020 to May 10, 2020), a total of 294 patients visited the emergency department with chest pain (vs. a mean of 572 in 2018-2019), that is, 48.6% fewer (p<0.05). The percentages gradually returned to normal, with no rebound effect or new easing of the second wave in October - November 2020. Similarly, during lockdown, we observed a decrease in the previous daily pattern of visits to the emergency room for chest pain that was noticed previously (higher number on workdays vs. weekends, p<0.05) (Figure 1B). This trend was re-established after lockdown.

### 3.2. Hospital admissions

A total of 497 patients were admitted with a diagnosis of IHD during 2020 (vs. an average of 606 during 2018-2019, that is, 18.0% fewer, p<0.05). In parallel to observations for the number of visits to the emergency department, we observed a reduction in the number of admissions due to IHD during lockdown (45 vs. 92 on average in 2018-2019, that is, 51.1% fewer, p<0.05), with recovery of the trend after lockdown and no rebound effect (Figure 1C). The percentage of admissions/visits to the emergency department during lockdown was 15.3% vs. 16.1% during 2018-2019 (p=NS), thus indicating that admission criteria were independent of the COVID-19 pandemic. These data point to an incident rate for IHD of 236.0 per 100,000 persons per year. Similarly, 29 of the patients who were admitted in 2020 had to be readmitted, that is, a rate of readmission per patient per year of 5.8% (vs. 2.3% for admissions during 2018-2019, p<0.05). Furthermore, as for the weekly change in admissions, significant differences were found for the 2018-2019 average in the number of cases of non–ST elevation–acute coronary syndrome (NSTE-ACS, ie, both NTSTEMI and unstable angina) admitted on weekdays (workdays) vs. weekends (p<0.05), with a larger number of patients in the former. During lockdown, we observed a reduction in the number of cases of ST elevation–acute coronary syndrome (STE- ACS) on weekdays (p<0.05), disappearance of the previous pattern of NSTEMI (not affected by the type of day during lockdown) and a significant reduction in the number of admissions for unstable angina during lockdown, both on weekdays and on weekends (p<0.05) (Figure 1D and Supplementary Figure 1).

The predominant profile of patients admitted with IHD in 2018-2019 was that of men in the seventh or eighth decade of life, with hypertension in two-thirds of cases, diabetes/dyslipidemia in around 50% of cases, chronic kidney disease (CKD) in up to one- third, and a history of IHD in up to 40%. When we compared admissions during lockdown, we observed a trend toward older age, with a higher percentage of octogenarians and history of diabetes and CKD, although patients tended not to have a history of IHD (Table 1) (p<0.05).

**Figure 2:**
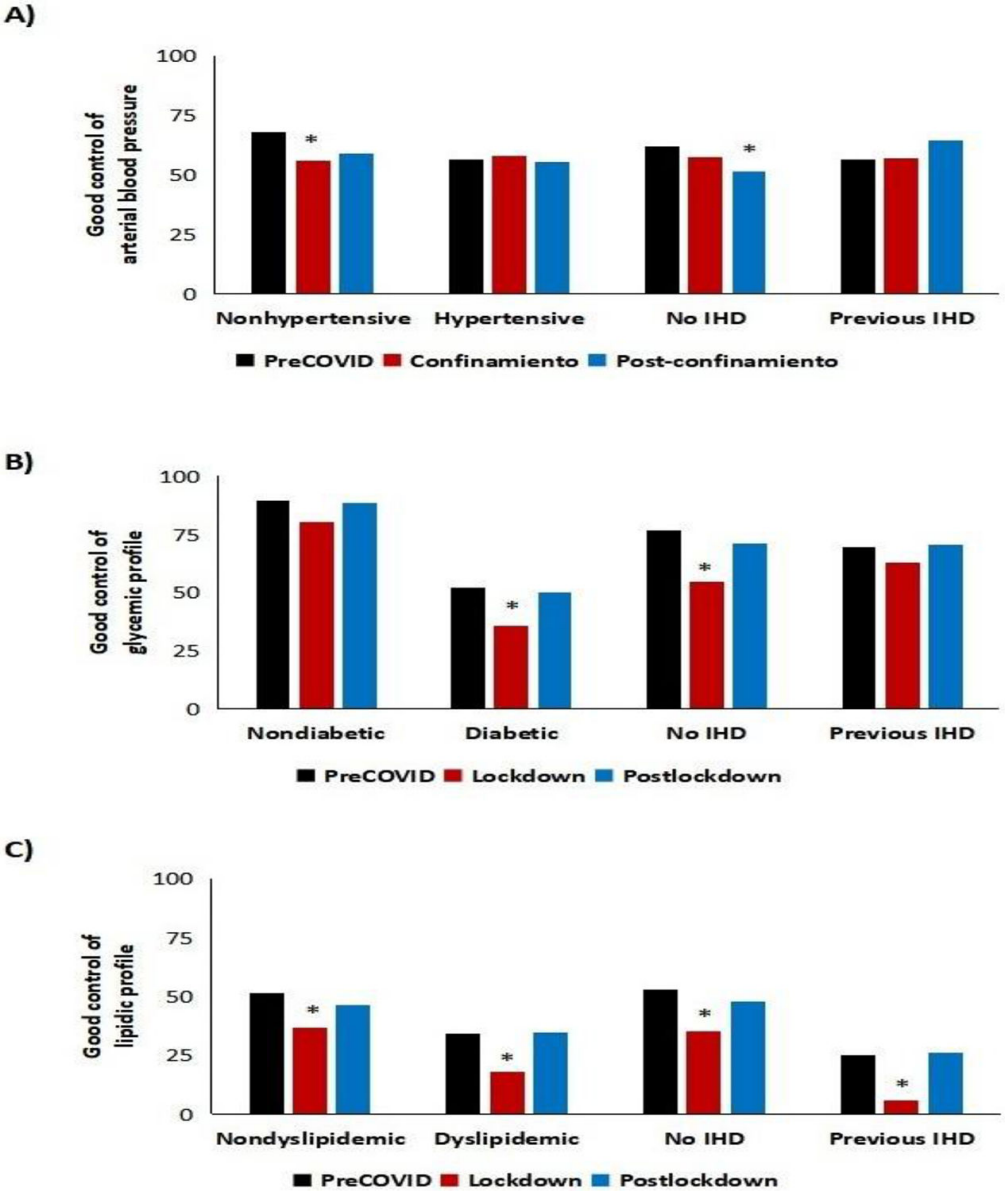
Rate of good control of Cardiovascular Risk Factors (CVRF). A) Arterial blood pressure. B) Blood glucose C) LDL-cholesterol. *p<0.05 vs. preCOVID and postlockdown. IHD, ischemic heart disease.

Depending on the type of IHD, we can observe multiple interactions (Supplementary Table 1), the most noteworthy of which are as follows: 1) Trends for age groups are confirmed; 2) During lockdown, patients who were admitted with STE-ACS were more frequently hypertensive than during the other periods and other types of ACS; 3) The prevalence of diabetes mellitus was higher since the lockdown for STEMI and unstable angina; 4) A personal history of heart disease was less frequent in patients who were admitted with unstable angina during lockdown; 5) The prevalence of CKD was higher during lockdown for patients admitted with acute myocardial infarction. Most parameters returned to normal after lockdown. The most notable aspect about the control of CVRFs is that it was suboptimal. In patients from 2018-2019, 58.7% had controlled hypertension, 52.3% had diabetes in range (10.8% diagnosed with diabetes during admission), and 41.1% had Low-Density Lipoprotein Cholesterol (LDLc) within targets. During lockdown, on the other hand, a higher percentage of patients had arterial hypertension (especially those with no personal history of arterial hypertension or IHD) (p<0.05), poorer glycemic control (p<0.05), and very poor lipid values, with only 5.7% of patients with IHD reaching their target (p<0.05) (Figure 2). Furthermore, the percentage of patients admitted during the preCOVID period with all CVRFs well controlled was 20.6%; during lockdown, this figure was only 9.5% (p<0.05), with a subsequent slight “normalization” after lockdown (17.6%).

**Figure 3:**
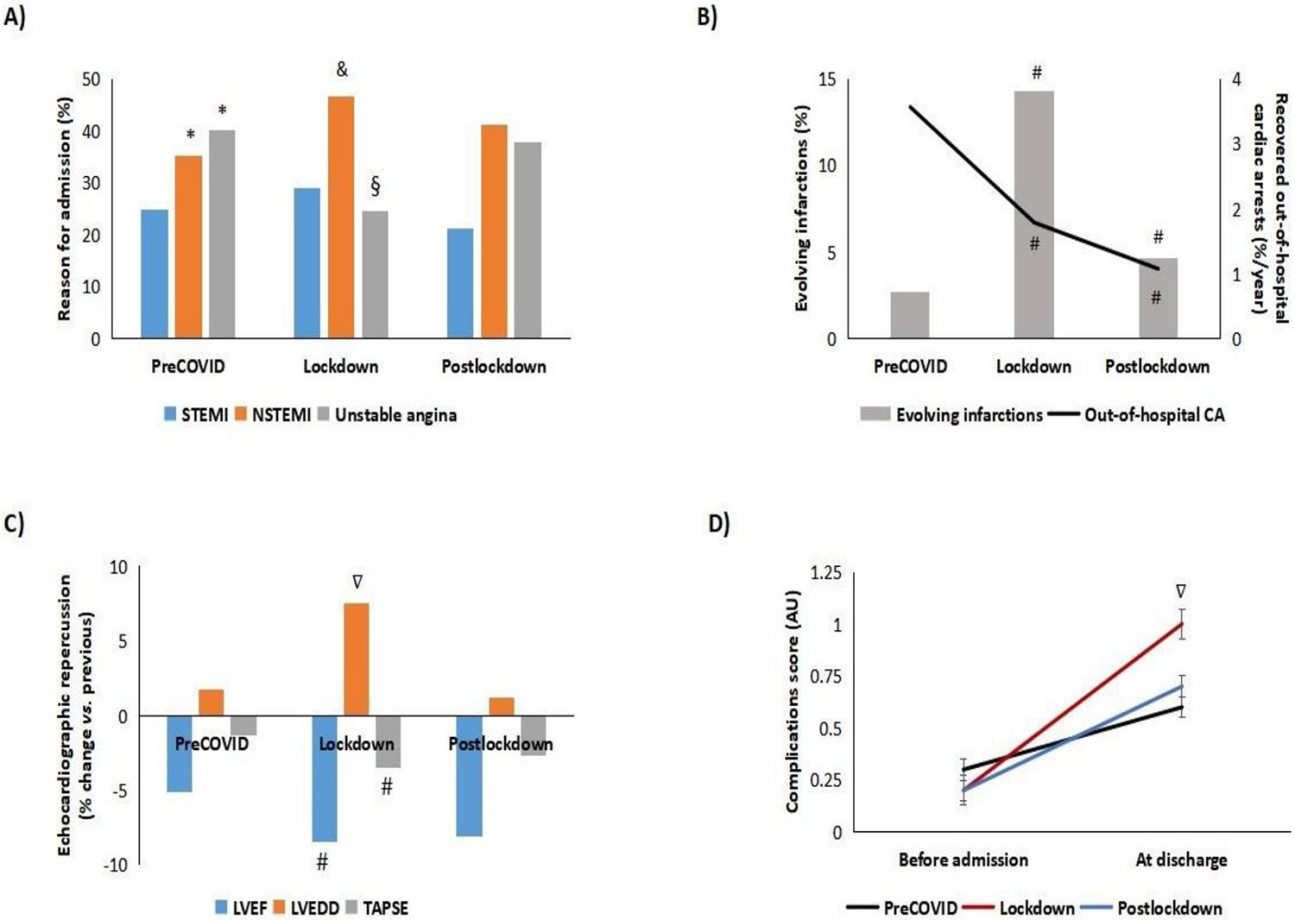
Characteristics of admissions and echocardiographic assessment of patients admitted for ischemic heart disease. A) Reason for admission. B) Cardiorespiratory arrest recovered outpatients and percentage of evolving infarcts. C) Variations of Left Ventricular Ejection Fraction (LVEF), Tricuspid Annular Plane Systolic Excursion (TAPSE) and Left Ventricular End-Diastolic Diameter (LVEDD) at admission vs. previous. D) Score of echocardiographic complications at discharge (see score in the Patients and Methods section). *p<0.05 vs. STEMI preCOVID. &p<0.05 vs. NSTEMI preCOVID. §p<0.05 vs. unstable angina from the preCOVID and postlockdown periods. #p<0.05 vs. preCOVID. ∇p<0.05 vs. preCOVID and postlockdown periods. AU, arbitrary units; NSTEMI, non–ST-Segment Myocardial Infarction; STEMI, ST-Segment Elevation Myocardial Infarction.

As for diagnosis at admission, during 2018-2019, the percentage of NSTE-ACS (both NSTEMI and unstable angina) was higher than that of STE-ACS (p<0.05) (Figure 3A). During lockdown, we observed a significant decrease in the percentage of unstable angina at the expense of an increase in NSTEMI (p<0.05). These variations returned to normal after lockdown. In an attempt to determine whether these results could be affected by a difference in the percentage of cases of out-of-hospital cardiac arrest that were eventually admitted to our center, we calculated the number of cases between 2018 and 2020 and found a significant decrease during lockdown (Figure 3B, p<0.05).

Furthermore, during lockdown, the percentage of cases of evolving infarctions admitted to hospital increased 5.3-fold compared with 2018-2019 (2.7% vs. 14.3%, p<0.05); this figure decreased after lockdown to reach values close to those recorded during the preCOVID era (4.7 %, p<0.05), while remaining statistical significance (Figure 3B).

Given the probable association with the abovementioned observation, we determined left ventricular ejection fraction (LVEF), left ventricular end-diastolic diameter LVEDD), and the tricuspid annular plane systolic excursion (TAPSE) at discharge (compared with previous echocardiographic data) and found poorer results during lockdown, with more pronounced decreases in LVEF and TAPSE and more cases of dilated left ventricle (p<0.05) (Figure 3C). Similarly, values on our in-house complications score (comprising ischemic dilated cardiomyopathy, grade of left ventricular systolic dysfunction, grade of left ventricular systolic dysfunction, right ventricular systolic dysfunction, ventricular aneurysm, and at least moderate mitral regurgitation) doubled in those patients who were admitted during lockdown compared with previous years (p<0.05) (Figure 3D). The frequency of interventional therapy (catheterizations/admission) during lockdown fell significantly with respect to NSTEMI in comparison with the preCOVID era (0.4 vs. 0.8, p<0.05). After lockdown rates recovered to “normal” (0.9).

**Figure 4:**
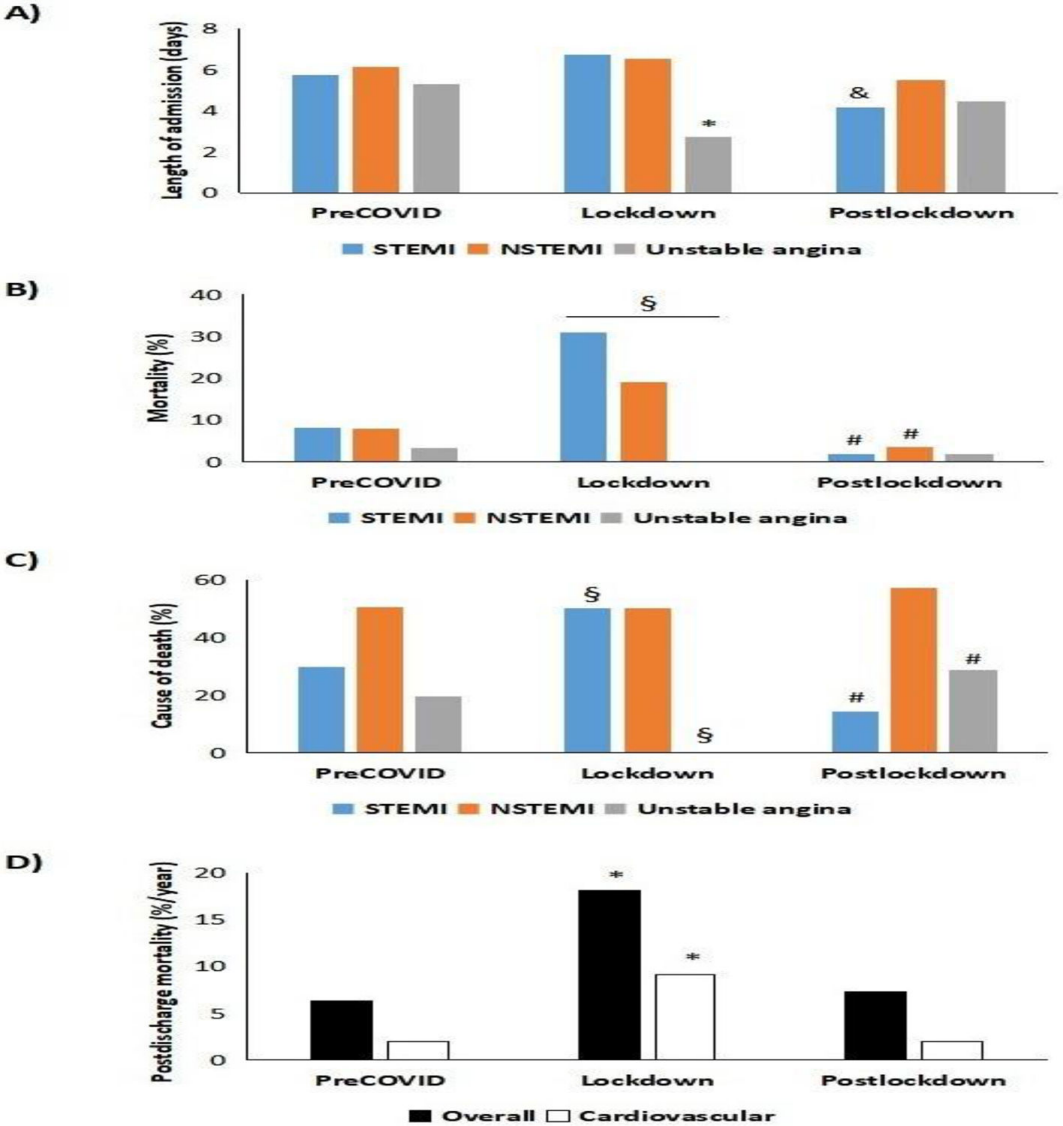
Mean stay and in- and out-hospital mortality. A) Length of admission B) In-hospital mortality C) Cause of deaths during admission D) Mortality outpatient after discharge * p<0.05 vs. unstable angina in the preCOVID period. &p<0.05 vs. STEMI in preCOVID period. §p<0.05 vs. preCOVID and post-confinement periods. #p<0.05 vs. preCOVID and confinement periods. NSTEMI, non-ST-segment elevation myocardial infarction; STEMI, ST-segment elevation myocardial infarction.

A breakdown by department reveals 3 relevant aspects: 1) In our center, the intensive care unit received (during the first 24 hours) 84.7% of cases of STEMI before 2020; during lockdown, this percentage fell by 55.6% thanks to support from the cardiology department (where this type of admission increased 6.2-fold) and from internal medicine (2.5-fold increase); 2) While the mean stay in the cardiology department decreased from 4.9 ± 0.4 days during 2018-2019 to 3.4 ± 0.3 days during lockdown (p<0.05), that of patients admitted to the intensive care unit increased from 1.2 ± 0.2 to 6.0 ± 0.2 days (with a return to normal values after lockdown) (p<0.05); and 3) In-hospital mortality according to the department patients were admitted to during lockdown varied dramatically in the intensive care unit, where it increased from 5.6% to 50.0%. In the case of the internal medicine department, in-hospital mortality increased from 27.5% to 36.4%, whereas it fell to zero for patients managed in the cardiology department (vs. 1.5% during the preCOVID period) (Supplementary Figure 2). Lastly, we analyzed the 2021 mortality rate for patients after a mean follow-up period of 691 days in the preCOVID group and 285 days for those who were admitted from the start of lockdown. In summary, both all-cause and cardiovascular mortality increased significantly among patients who were discharged after having been admitted during lockdown (p<0.05) (Figure 4D and Supplementary Table 2B). Cardiovascular mortality accounted for 30.2% of all-cause mortality among those patients who were admitted before the COVID-19 pandemic. However, this figure increased to 49.7% (p<0.05) for patients who were admitted during lockdown and then fell to 26.0% after lockdown (p<0.05). It is also worth noting that of the 18 deaths among patients who were admitted during lockdown, 55% died during admission (vs. <25% during the preCOVID period and <10% after lockdown).

## 4. Discussion

The COVID-19 pandemic generated a major health problem at the beginning of 2020. Such was the need to centralize resources around SARS-CoV-2 infection that it relegated other conditions (e.g., IHD and cancer) to second place. Reduced attendance at care centers and underdiagnosis of diseases that, owing to their pathophysiology, continued to appear during the “first wave” were probably the result of fear of contracting the virus, lockdown itself, the idea of not overloading hospitals with non-COVID diseases, the redistribution of health professionals outside their specialties, and the key role played by the pandemic itself (competitive risk) [5]. The present study highlights some of these consequences. First, an almost 50% decrease in the number of visits to the emergency department for chest pain (or similar) was recorded, with a resulting decrease in the number of admissions for IHD (of similar magnitude). These results are consistent with those published by other research groups [3-7] and with data from the European Society of Cardiology during lockdown [8]. Given that the decrease was not offset by a subsequent increase in the number of admissions for IHD, the incidence of coronary disease may have been similar, although some patients may have remained in their homes. This could be a consequence of the increase in the number of cases of out-of-hospital cardiac arrest in our series and in several European studies [9,10], probably owing to the emotional stress of the pandemic (e.g., isolation, loneliness, depression, anxiety over employment) and its higher fatality rates (owing to the absence of someone to warn or help and the greater delay in receiving medical care). The first hypothesis would account for the decrease in admissions for unstable angina and the greater percentage of evolving infarctions that reached our hospital (more than 5-fold greater than during the preCOVID period). Furthermore, during the preCOVID era, we observed a weekly pattern of visits to the emergency department and admissions, with NSTE-ACS being significantly more common on Mondays, falling gradually to become significantly less frequent at the weekend (Supplementary Figure 1, upper panel). However, this trend disappeared during lockdown (Supplementary Figure 1, middle panel). One plausible explanation could be that of a heightened catecholaminergic state on the first days of the week (greater psychosocial stress), as recently shown in the Swedish registry study SWEDEHEART [11]. With lockdown, any potential stress would have been spread throughout the week, and with no possibility of going to work from Monday to Friday, this pressure would decrease, as would, presumably, the incidence of IHD.

Another important aspect of this study was that of updating the real profile of a patient experiencing IHD for the first time, both in recent years (preCOVID) and after lockdown. The typical patient was male (2:1), at the end of the seventh decade of life, with hypertension (≈70%), diabetes (≈40%), dyslipidemia (≈60%), CKD (>20%), and a history of IHD (up to 40%). More octogenarians were admitted during lockdown: fewer had a history of cardiovascular disease, although more had CVRFs, thus indicating that they were more likely to experience a first cardiovascular event. A more in-depth analysis of control of CVRFs reveals that during 2018-2019, the conclusions of the pivotal EUROASPIRE V and DA VINCI studies are confirmed [12,13], namely, uncontrolled arterial blood pressure in more than 40% of patients (known hypertension or not), blood sugar levels in range in <55% of diabetic patients (with >10% of cases of diabetes diagnosed at admission), and LDLc out of range in >65% of patients with dyslipidemia (of note, in 49% of seemingly healthy patients). During lockdown, significantly impaired glycemic control was observed in diabetic patients and in patients with heart disease. The same was true of lipid control, which worsened significantly in all the groups studied. Such was the situation that during lockdown, only 9.5% of patients had all CVRFs controlled (vs. 20.6 and 17.6% during the preCOVID and postlockdown periods, respectively). A study of the correlation between CVRFs and mortality enables us to conclude the following: 1) Being older than 65 years and having hypertension or CKD was associated with greater mortality (all-cause and cardiovascular); 2) Mortality increased overall during lockdown, although this was more marked in the groups mentioned; and 3) CKD and diabetes mellitus were associated with double the risk of cardiovascular death during lockdown. In addition to the association between CVRFs and mortality, lockdown was characterized by more cases of evolving infarction, with poorer residual LVEF, lower TAPSE , more cases of dilated left ventricle, and a greater number of structural complications. These findings may have contributed to greater mortality both in hospital (three-fold higher vs. preCOVID) and after discharge (four times more, especially cardiovascular mortality). These data are consistent with those of the Interventional Cardiology Association of the Spanish Society of Cardiology [4]. We recorded fewer catheterizations during lockdown (40% fewer), especially with respect to NSTEMI. However, patients who underwent percutaneous coronary intervention received stents more frequently than during the preCOVID period, thus potentially implying better selection of candidates. Among limitations of this research we consider the modest number of patients who attended the Hospital during lockdown and the lack of baseline echocardiograms in some patients (less than 10%), what may affect the accuracy of some substudies.

## 5. Conclusion

In conclusion, this study confirms a decrease in the number of admissions for IHD during the COVID-19 lockdown and a lower number of cases of out-of-hospital cardiac arrest that were subsequently admitted to our hospital. Our study also provides information on the profile of patients who were admitted to hospital. These patients were characterized by poor control of CVRFs, more frequent evolving infarction and echocardiographic complications, and greater mortality both at admission and after discharge. Most of the parameters studied returned to normal values after lockdown, thus leading us to believe that there may be a causal relationship between these findings and lockdown itself beyond SARS-CoV-2 infection (especially when the greater incidence and mortality of COVID-19 in our area shifted to the months of September - December). These data, which we consider can be extrapolated to other health areas, should lead us to reflect and increase our knowledge, as well as to consider how we can manage our resources in future situations where mobility is limited to the same extent.

## Supplementary Material

1

## Figures and Tables

**Table 1:** Baseline clinical characteristics. Values are expresses as mean ± standard deviation for age and percentage (95% confidence interval) for the rest of parameters. AHT: Arterial Hypertension; CKD: Chronic Kidney Disease; DLP: Dyslipidemia; DM: Diabetes Mellitus; IHD: Ischemic Heart Disease; NS: Nonsignificant.

	PreCOVID	Lockdown	Postlockdown	Significance
	(n=1,301)	(n=45)	(n=343)	(p)
Age (years)	69.3 ± 12.0	72.1 ± 12.8	68.7 ± 12.2	NS
>80 years	22.5 (20.2 - 24.8)	33.9 (20.1 - 47.7)	22.2 (17.8 - 26.6)	<0.05
Women	31.8 (29.3 - 34.3)	28.9 (15.7 - 42.1)	38.2 (33.1 - 43.3)	<0.05
AHT	73.0 (70.6 - 75.4)	67.4 (53.7 - 81.1)	71.5 (66.7 - 76.3)	NS
DM	43.7 (41.0 - 46.4)	55.3 (40.8 - 69.8)	46.2 (40.9 - 51.5)	<0.05
DLP	57.4 (54.7 - 60.1)	58.7 (44.3 - 73.1)	59.3 (54.1 - 64.5)	NS
IHD	39.5 (36.8 - 42.2)	26.1 (13.3 - 38.9)	40.0 (34.8 - 45.2)	<0.05
CKD	23.3 (21.0 - 25.6)	33.3 (19.5 - 47.1)	17.5 (13.5 - 21.5)	<0.05
AHT + DM	38.4 (35.8 - 41.0)	47.8 (33.2 - 62.4)	40.0 (34.8 - 45.2)	<0.05
AHT + DLP	46.1 (43.4 - 48.8)	41.3 (26.9 - 55.7)	50.2 (44.9 - 55.5)	<0.05
AHT + CI	33.2 (30.6 - 35.8)	21.7 (9.7 - 33.7)	34.1 (29.1 - 39.1)	<0.05
DM + DLP	30.6 (28.1 - 33.1)	41.3 (26.9 - 55.7)	35.7 (30.6 - 40.8)	<0.05
DM + IHD	22.9 (20.6 - 25.2)	15.2 (4.7 - 25.7)	23.9 (19.4 - 28.4)	NS
DLP + IHD	29.9 (27.4 - 32.4)	21.7 (9.7 - 33.7)	31.1 (26.2 - 36.0)	NS
AHT + DM + DLP	27.2 (24.8 - 29.6)	34.8 (20.9 - 48.7)	33.1 (28.1 - 38.1)	NS
AHT + DM + IHD	21.1 (18.9 - 23.3)	15.2 (4.7 - 25.7)	22.3 (17.9 - 26.7)	NS
AHT + DLP + IHD	26.0 (23.6 - 28.4)	19.6 (8.0 - 31.2)	28.2 (23.4 - 33.0)	NS
DM + DLP + IHD	18.1 (16.0 - 20.2)	15.2 (4.7 - 25.7)	21.0 (16.7 - 25.3)	NS
AHT + DM + DLP + IHD	17.0 (15.0 - 19.0)	15.2 (4.7 - 25.7)	20.3 (16.0 - 24.6)	NS

**Table 2:** Mortality according to cardiovascular risk factors. The data presented show the monthly percentage for all-cause mortality and, in parenthesis, cardiovascular mortality.

	PreCOVID (n=1,301)	Lockdown (n=45)	Postlockdown (n=343)	Significance (p)
**Age**				
**>65 years**	2.0 (1.0)	24.3 (17.4)	1.8 (0.7)	<0.05
**<65 years**	0.2 (0.1)	0.0 (0.0)	0.0 (0.0)	<0.05
**Arterial hypertension**				
**Yes**	2.8 (0.8)	18.5 (12.9)	1.3 (0.4)	<0.05
**No**	0.4 (0.3)	14.8 (11.1)	0.8 (0.5)	<0.05
**Diabetes mellitus**				
**Yes**	1.8 (0.7)	18.5 (16.2)	1.0 (0.4)	<0.05
**No**	1.1 (0.6)	15.9 (7.9)	1.2 (0.4)	<0.05
**Dyslipidemia**				
**Yes**	1.5 (0.7)	17.1 (10.7)	0.8 (0.4)	<0.05
**No**	1.3 (0.6)	17.5 (14.6)	1.3 (0.5)	<0.05
**Chronic kidney disease**				
**Yes**	2.9 (1.4)	25.9 (18.5)	2.4 (0.3)	<0.05
**No**	0.8 (0.4)	13.0 (9.3)	0.9 (0.5)	<0.05

## Abbreviations:

AHT: Arterial Hypertension
ACS: Acute Coronary Syndrome
CKD: Chronic Kidney Disease
CVRFs: Cardiovascular Risk Factors
DLP: Dyslipidemia
DM: Diabetes Mellitus
IHD: Ischemic Heart Disease
LDLc: Low-Density Lipoprotein Cholesterol
LVEDD: Left Ventricular End-Diastolic Diameter
LVEF: Left Ventricular Ejection Fraction
NSTEMI: Non ST-Segment Elevation Myocardial Infarction
PCI: Percutaneous Coronary Intervention
SARS-CoV-2: Severe Acute Respiratory Syndrome Coronavirus 2
STEMI: ST-Segment Elevation Myocardial Infarction
TAPSE: Tricuspid Annular Plane Systolic Excursion
