# Supplementary material for: Impact of Coronavirus-19 Pandemic and Lockdown on Admissions for Ischemic Heart Disease
Source: Cardiol Cardiovasc Med. Author manuscript; Available in PMC 2023 Mar 23. (PMC10035782; doi:10.26502/fccm.92920270)
Supplement: 1 [file NIHMS1880234-supplement-1.pdf]

Supplementary Files:

	STEMI			NSTEMI			Unstable angina			Significance (p)
	18-19	LD	Post-LD	18-19	LD	Post-LD	18-19	LD	Post-LD	
Age (years)	64.1	66.8	64.4	71.9	76.2	70.3	68.6	71.5	67	<0.05
AHT (%)	56.6	69.2	60	76.6	66.7	71.9	79.9	66.7	77.7	<0.05
DM (%)	35.2	53.8	36.9	51.3	47.6	46.1	42.1	66.7	51.8	<0.05
DLP (%)	47.7	38.5	46.2	60.9	66.7	57.8	60.2	66.7	68.8	<0.05
IHD (%)	19.4	15.4	20	39.6	33.3	39.8	52.1	25	51.8	<0.05
CKD (%)	8.2	23.1	10.2	32.3	47.6	19	26.2	18.2	20	<0.05
AHT + DM (%)	27.2	53.8	29.2	46.4	42.9	39.8	38.3	50	46.4	<0.05
AHT + DLP (%)	28.5	23.1	29.2	50.8	47.6	50	52.9	50	62.5	<0.05
AHT + IHD (%)	13.9	15.4	18.5	33.5	23.8	35.9	44.7	25	41.1	<0.05
DM + DLP (%)	21.1	23.1	26.2	36.1	42.9	35.9	31.4	58.3	41.1	<0.05
DM + IHD (%)	9.1	15.4	13.8	27.6	14.3	22.7	27.4	16.7	31.3	<0.05
DLP + IHD (%)	14	15.4	10.8	32.9	23.8	32.8	37.2	25	41.1	<0.05
AHT + DM + DLP (%)	15.1	23.1	20	33	38.1	34.4	29.5	41.7	39.3	<0.05
AHT + DM + IHD (%)	8.5	15.4	12.3	25.4	14.3	21.9	25.1	16.7	28.6	<0.05
AHT + DLP + IHD (%)	10.5	15.4	9.2	28.3	19	30.5	33.7	25	36.6	<0.05
DM + DLP + IHD (%)	6.8	15.4	10.8	22.5	14.3	21.1	21.3	16.7	26.8	<0.05
AHT + DM + DLP +	6.1	15.	9.2	20.9	14.	21.1	20.3	16.	25.9	<0.05

IHD (%)		4			3			7		

Table 1: Baseline clinical characteristics. Values are expressed as a percentage or mean ± standard deviation. AHT: Arterial Hypertension; CKD: Chronic Kidney Disease; DLP: Dyslipidemia; DM: Diabetes Mellitus; IHD: Ischemic Heart Disease; LD: Lockdown; NS: Nonsignificant.

**A**	**PreCOVID (n=1,301)**			**Lockdown (n=45)**			**Postlockdown (n=343)**			**Significance (p)**
	**STEMI**	**NSTEMI**	**UA**	**STEMI**	**NSTEMI**	**UA**	**STEMI**	**NSTEMI**	**UA**	
Cardiology	0.2	0.9	0.4	0	0	0	0	0.4	0.4	<0.05
ICU	4.2	0.3	0.1	37.5	12.5	0	0	0	0	<0.05
IM	7.2	14.5	5.8	9.1	27.3	0	3.8	11.5	1	<0.05
B	PreCOVID (n=1,301)			Lockdown (n=45)			Postlock down (n=343)			Significance (p)
All-cause mortality (%)	11.7			15.4			5.6			<0.05
All-cause mortality (%/year)	6.3			18.1			7.3			<0.05
Cardiovascular death (%)	3.5			7.7			1.5			<0.05
Cardiovascular death (%/year)	1.9			9			1.9			<0.05

Table 2: Mortality rates A) During admission by department and type of ACS B) Postdischarge mortality. Data are expressed as percentages. STEMI, ST- Segment Elevation Myocardial Infarction; NSTEMI, Non–ST-Segment Elevation Myocardial Infarction; UA, Unstable Angina; ICU, Intensive Care Unit; IM, Internal Medicine.

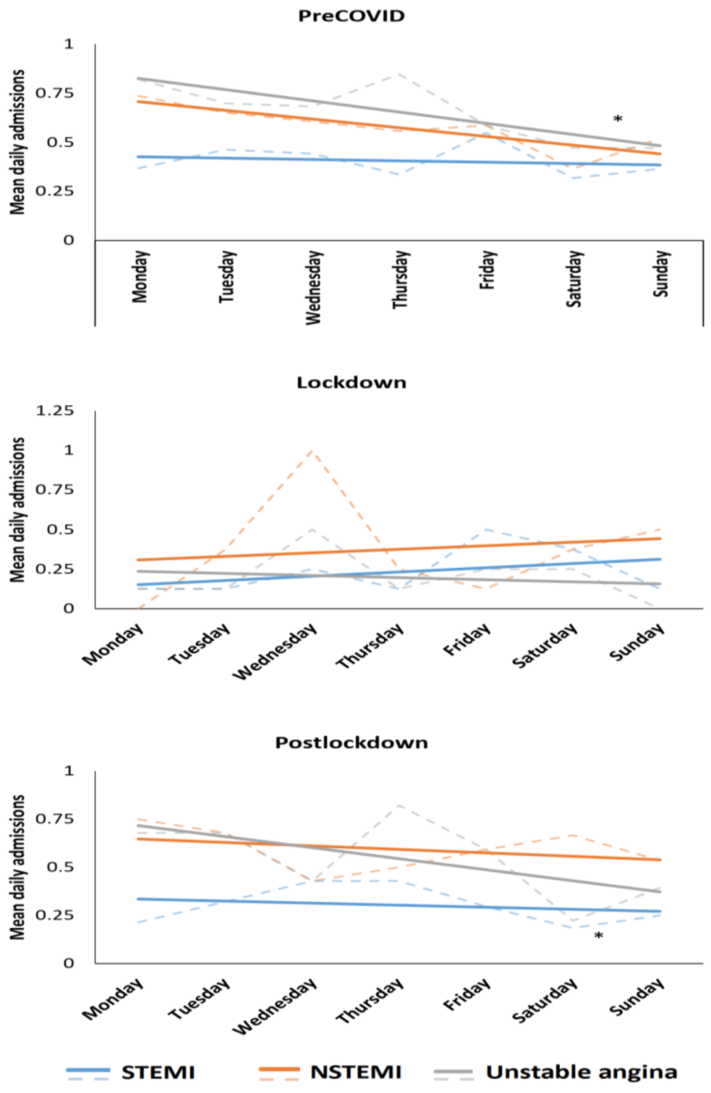

Figure 1: Daily admissions for ischemic heart disease. Weekly profile of admissions for the different types of ACS. *p<0.05 weekends vs. weekdays. STEMI, ST - Segment Elevation Myocardial Infarction; NSTEMI, non-ST-Segment Myocardial Infraction.

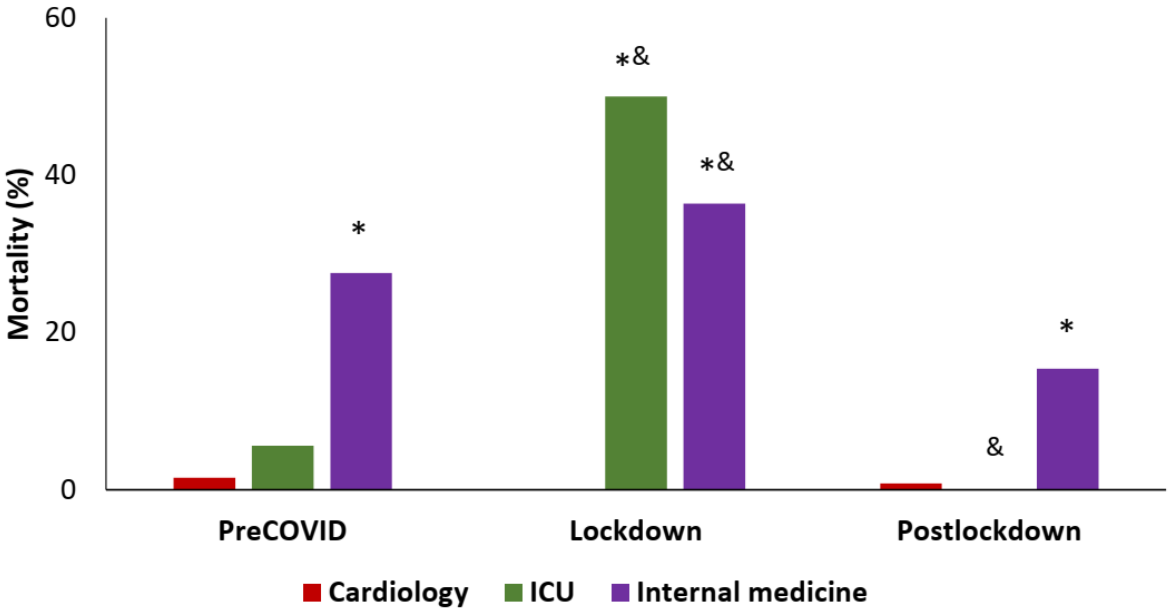

Figure 2: In-hospital mortality by department. *p<0.05 vs. other departments for the same period. &p<0.05 vs. same department for other periods. ICU: Intensive Care Unit.
